# Surface Properties, Color Stability, and Bacterial Adhesion of Implant‐Supported Provisional Restorative Materials: In Vitro Study

**DOI:** 10.1002/cre2.70461

**Published:** 2026-10-12

**Authors:** Júlia Bárbara Medeiros Galvão, Richelle Nunes Costa, Aliane da Silva Bezerra, Liliane Cristina Nogueira Marinho, Ana Luísa de Barros Pascoal, Pedro Henrique Sette de Souza, André Ulisses Dantas Batista, Patrícia dos Santos Calderon

**Affiliations:** ^1^ Department of Dentistry Federal University of Rio Grande do Norte Natal Brazil; ^2^ Department of Dentistry University of Pernambuco Arcoverde Brazil; ^3^ Department of Restorative Dentistry Federal University of Paraíba João Pessoa Brazil

**Keywords:** bacterial adhesion, color, surface properties, wettability

## Abstract

**Objectives:**

To evaluate surface roughness, wettability, color stability, and bacterial adhesion of different materials used in implant‐supported provisional restorations.

**Materials and Methods:**

Eighty samples (6 × 6 × 2 mm ± 0.2 mm) were fabricated and distributed into four groups (*n* = 20): Bis‐acryl composite resin (BCR); CAD‐CAM PMMA resin (Milled); Auto‐polymerized acrylic resin (APR); prefabricated acrylic tooth (PT). Surface roughness was measured using a 3D optical profilometer, wettability by the sessile drop method, and color stability with a spectrophotometer after immersion in distilled water and coffee. Bacterial adhesion was assessed through *Streptococcus mutans* biofilm formation and quantified spectrophotometrically after crystal violet staining. Data were analyzed using Kruskal–Wallis and one‐way ANOVA with Tukey's post hoc test (*α* = 0.05).

**Results:**

Bis‐acryl composite resin (BCR) showed the lowest surface roughness, while prefabricated acrylic tooth (PT) exhibited higher roughness and contact angle values (*p* < 0.001) and the greatest color change (Δ*E* ≥ 3.3). BCR demonstrated the highest bacterial adhesion, whereas CAD‐CAM PMMA resin (Milled) showed the lowest adhesion.

**Conclusions:**

The behavior of provisional restorative materials is influenced by their composition, affecting surface, optical, and biological performance. Lower surface roughness did not result in reduced biofilm formation. CAD/CAM‐milled and Auto‐polymerized resins showed more favorable overall performance.

## 1 Introduction

1.1

Temporary restorations on implants are crucial for improving the esthetics, contour, and shaping of the peri‐implant tissue architecture (Ribera et al. 2023; Sahin et al. 2025). Restorations must meet mechanical, biological, and esthetic requirements compatible with the oral environment (Bandarra et al. 2020; Gantz et al. 2021). To achieve these goals, materials must withstand different intraoral conditions, including thermal changes, high humidity, and repetitive occlusal loads, for a clinically relevant period (Reeponmaha et al. 2020).

Mechanical integrity is particularly important, as fracture or deformation may compromise treatment outcomes and require additional clinical interventions (Astudillo‐Rubio et al. 2018; Sousa‐Santos et al. 2023). In implant‐supported rehabilitations, provisional restorations frequently remain in function for extended periods during osseointegration and peri‐implant tissue conditioning. Under these conditions, interim materials must demonstrate mechanical durability, wear resistance, and biocompatibility (Gantz et al. 2021; Haralur et al. 2024).

Provisional restorations may be fabricated using direct or indirect techniques, with Auto‐polymerized acrylic resin and bis‐acrylic resins widely used due to their cost‐effectiveness and clinical practicality (Soares et al. 2019; Bitencourt et al. 2020). However, Auto‐polymerized acrylic resin may present polymerization shrinkage, dimensional instability, residual monomer release, and increased surface roughness (Reeponmaha et al. 2020). CAD/CAM‐milled materials, produced from pre‐polymerized polymers, demonstrate improved mechanical properties and color stability, although fabrication limitations and material waste remain concerns (Ribera et al. 2023; Taşın et al. 2022; Giti et al. 2021).

Beyond mechanical performance, esthetic and biological stability are critical for provisional restorations (Song et al. 2020). Color change (Δ*E*) may negatively affect patient perception, particularly in esthetic areas, and is influenced by intrinsic material composition and extrinsic factors such as water absorption and dietary pigments (Bitencourt et al. 2020). Prolonged use may also favor bacterial colonization, especially when surface irregularities facilitate biofilm accumulation (Giti et al. 2021; Souza et al. 2020). In this context, finishing and polishing protocols play a decisive role in determining surface quality, directly affecting optical behavior and microbial retention (Gantz et al. 2021; Haralur et al. 2024; Hashemzade et al. 2025). Nevertheless, evidence integrating surface characteristics with color stability and bacterial adhesion remains limited.

Despite the widespread use of different materials for implant‐supported provisional restorations, their overall surface and biological behavior remain insufficiently characterized. Surface roughness, wettability, color stability, and bacterial adhesion are interrelated and may influence the clinical performance of provisional restorations. This is particularly relevant when provisional restorations are maintained for extended periods during soft‐tissue conditioning, as commonly occurs in implant therapy in the esthetic area. However, evidence comparing these properties across different provisional restorative materials remains limited. Therefore, this study aimed to evaluate the surface roughness, wettability, color stability, and bacterial adhesion of different implant‐supported provisional restorative materials. The study hypothesis was that the different materials would exhibit significant differences in surface properties.

## 2 Materials and Methods

### 2.1 Study Design

Eighty samples were prepared and allocated into four experimental groups (*n* = 20) according to the provisional restorative material: Bis‐acryl composite resin (BCR); CAD‐CAM PMMA resin (Milled); Auto‐polymerized acrylic resin (APR); prefabricated acrylic tooth (PT) (Figure 1). Specimens were fabricated in a standardized block shape measuring approximately 6 × 6 × 2 mm (± 0.2 mm). The materials used in this study are listed in Table 1, including their commercial names, manufacturers, composition, and batch numbers.

**Figure 1:**
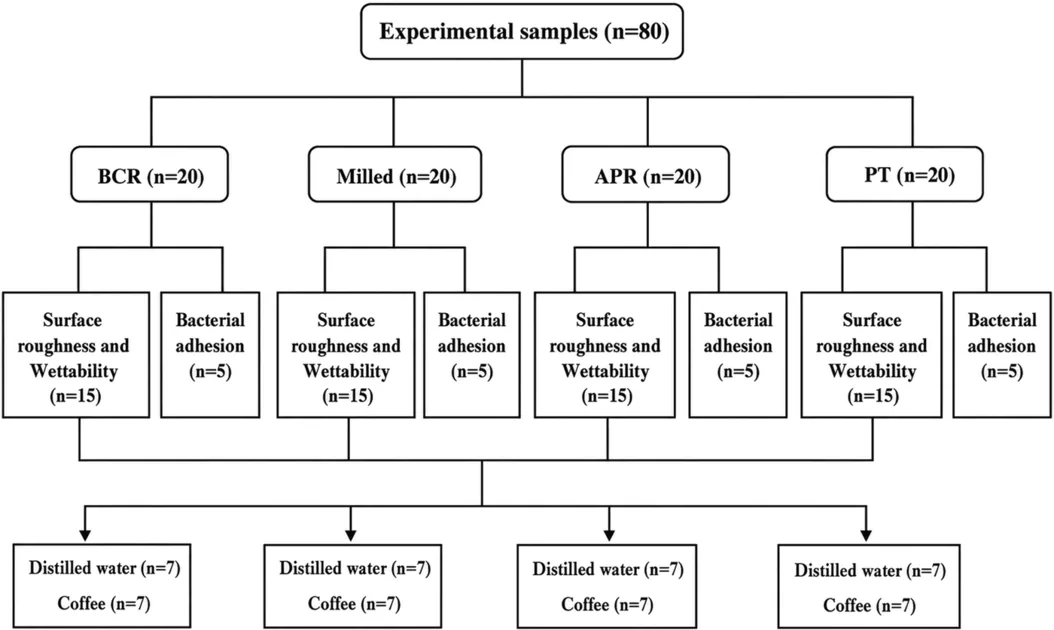
Schematic drawing of experimental groups. APR, Auto‐polymerized acrylic resin; BCR, bis‐acryl composite resin; Milled, CAD‐CAM PMMA resin; PT, prefabricated acrylic tooth.

**Table 1:** Material, trade name, manufacturer, composition, and batch number.

Material	Trade name	Manufacturer	Composition	Batch
Auto‐polymerized acrylic resin	VipiCor	VIPI Industry, Commerce, Export and Import of Dental Products Ltd., Pirassununga, São Paulo, Brazil	Polymethacrylate, Benzoyl Peroxide and Biocompatible Pigments.	0000118041
Self‐curing acrylic liquid	VIPIFLAH SH	VIPI Industry, Commerce, Export and Import of Dental Products Ltd., Pirassununga, São Paulo, Brazil	Methyl methacrylate, DMT, Inhibitor, EDEMA and Fluorescent.	0000162908
Bis‐acryl composite resin	Protemp 4	3M ESPE, Sumaré, São Paulo, Brazil	BASE PASTE: Dimethacrylate (BISEMA6), amorphous silica treated with silane, polyurethane methacrylate, silica treated with silane.	8350387
			CATALYZED PASTE: RA:Ethanol,2,2’[(1methylethylidene), bis (4‐phenylenoxy) bis, diacetate, silica treated with silane, benzyphenylbarbituric acid.	
CAD‐CAM PMMA resin	Vipiblock	TRILUX VIPI Industry, Trade, Export and Import of Dental Products Ltd., Pirassununga, São Paulo, Brazil	Polymethacrylate, Biocompatible Pigments, Fluorescent and EDMA.	0000099225
Prefabricated acrylic tooth	Dentute Teeth	VIPI Industry, Commerce, Export and Import of Dental Products Ltd., Pirassununga, São Paulo, Brazil	Polymethyl methacrylate	0000022021
			Dimethacrylate‐fluorescent pigments—ceramic‐organic modified.	

### 2.2 Sample Preparation

A digital caliper (Nove54) was used to ensure dimensional accuracy and standardization of all specimens. For the CAD‐CAM PMMA resin (Milled) and Prefabricated acrylic tooth (PT), block‐shaped specimens were sectioned from a PMMA CAD/CAM block (Vipiblock, VIPI, Pirassununga, São Paulo, Brazil) and prefabricated acrylic tooth (PT) (Dentute Teeth, Pirassununga, São Paulo, Brazil), respectively, using a double‐sided diamond disc (American Burrs, Palhoça, Santa Catarina, Brazil) coupled to a micromotor (Talmax, Dental Prosthetics Products, Paraná, Brazil). For the Auto‐polymerized acrylic resin (APR) and Bis‐acryl composite resin (BCR) groups, molds were fabricated using condensation silicone (Zetaplus, Zhermack, Badia Polesine, Italy) according to the predetermined dimensions (6 × 6 × 2 mm).

For the Auto‐polymerized acrylic resin (APR), the powder and liquid components (VipiCor and VIPI Flash, VIPI, Pirassununga, São Paulo, Brazil) were mixed in a glass dappen dish at a 3:1 ratio (by weight), using a precision balance, according to the manufacturer's instructions. The mixture was inserted into the mold to completely fill the cavity, and a glass slab coated with petroleum jelly was placed over the mold to obtain a flat and standardized surface. The same molds were used for fabrication of the Bis‐acryl composite resin (BCR) (Protemp 4, 3M ESPE, Sumaré, São Paulo, Brazil). The material was dispensed directly into the molds using the automixing cartridge tip for up to 40 s, following the manufacturer's recommended working time. A lubricated glass slab was then placed over the mold until complete polymerization.

After fabrication, all specimens were sequentially polished using silicon carbide abrasive papers (#220, #800, #1000, and #1200 grit; 3M, St. Paul, MN, USA) in a metallographic polishing machine (PVV, Teclago, São Paulo, Brazil), standardizing a polishing time of 10 s per surface.

The specimens underwent finishing and polishing procedures according to the material group. For the Auto‐polymerized acrylic resin (APR) and prefabricated acrylic tooth (PT) groups, a sequence of polishing rubbers from a finishing and polishing kit (American Burrs, Palhoça, Santa Catarina, Brazil) was used, followed by polishing with goat‐hair brushes attached to a micromotor. For the Bis‐acryl composite resin (BCR), the specimens were cleaned with 70% ethanol (Flop's 70; Tangará Química, Pederneiras, São Paulo, Brazil) without additional polishing procedures. The CAD‐CAM PMMA resin group (Milled) did not receive any finishing or polishing treatment after sectioning.

### 2.3 Surface Roughness

Fifteen samples per group were subjected to surface topography analysis for roughness (*R*_a_) measurement using a non‐contact 3D optical profilometer (CCI MP, Taylor Hobson, Leicester, UK), connected to a computerized unit equipped with Talysurf CCI software (Taylor Hobson, UK) for data acquisition. A cutoff value of 0.25 mm was applied, using a 50*×* objective lens with a numerical aperture of 0.4 and a scanning speed of ×3 in xyz acquisition mode (resolution: 516 × 516 pixels). Three measurements were performed on each specimen, and the mean *R*_a_ value was calculated for statistical analysis.

### 2.4 Wettability

Wettability was assessed by measuring the water contact angle (*θ*C) using the sessile drop method with distilled water. A 10‐µL droplet of distilled water (Automotive Company, São Paulo, Brazil) was dispensed onto the specimen surface using an automatic micropipette, positioning the tip as close as possible to avoid impact distortion (Figure 2).

**Figure 2:**
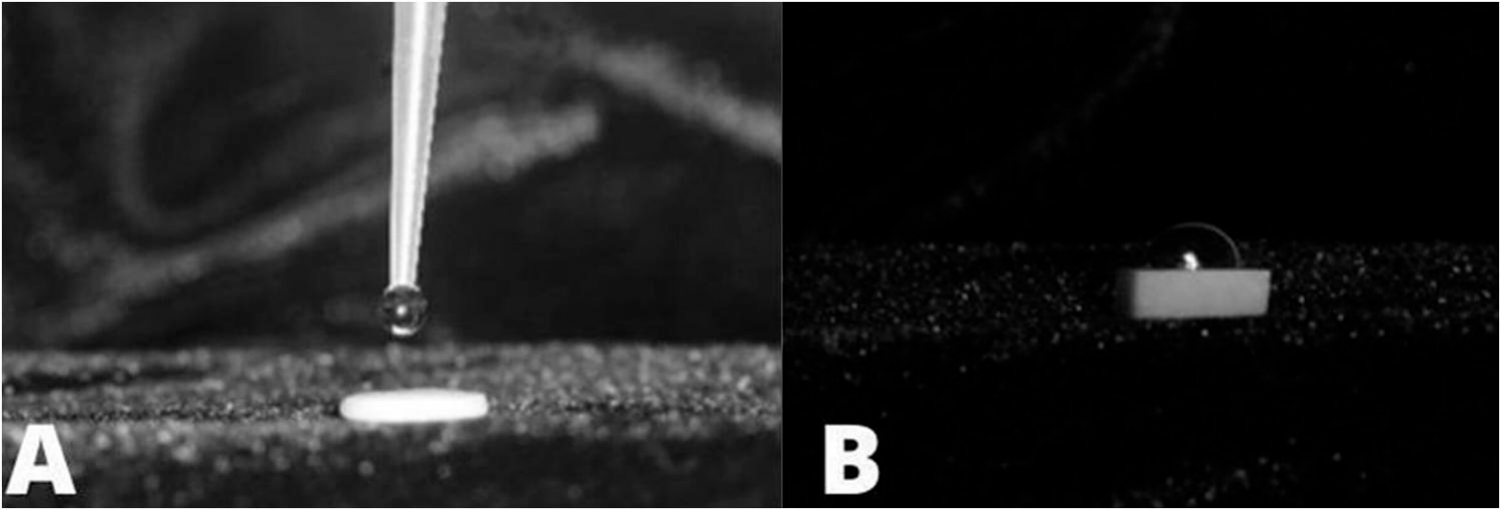
Representative image of the sessile drop being deposited onto the surface of the sample for contact angle measurement. (A) pipette; (B) image standard used in analyses.

The droplet image was captured 5 s after contact using a digital camera (Canon, Tokyo, Japan) mounted on a tripod at a standardized distance of 30 cm from the specimen. Contact angle measurements (degrees) were analyzed using dedicated software (Surftens 4.5, OEG GmbH, Germany). Three measurements were obtained per specimen, and the mean value was calculated for statistical analysis. Surface free energy was inferred based on the contact angle measurements. Group values were determined from the means of individual specimen measurements.

### 2.5 Color Stability Analysis

Color stability was evaluated using the CIELab* system. Fifty‐six specimens (*n* = 14 per group) were analyzed, with seven specimens immersed in distilled water (Automotive Company, São Paulo, Brazil) and seven in coffee solutions (Santa Clara Clássico, 3 Corações Alimentos S.A., Eusébio, Ceará, Brazil). Baseline color measurements were obtained using a spectrophotometer (VITA Easyshade V, VITA Zahnfabrik, Bad Säckingen, Germany) after cleaning the specimens with 70% alcohol (Flop's 70; Tangará Química, Pederneiras, São Paulo, Brazil) and drying with absorbent paper (Nalgene Versi‐Dry, Thermo Fisher Scientific, Rochester, NY, USA). All readings were performed against a white background by a single calibrated operator.

The coffee solution was prepared by dissolving 1 g of instant coffee in 50 mL of boiling mineral water. Specimens were immersed in 5 mL of either coffee or distilled water and stored at 37°C. Solutions were renewed every 24 h for 7 days. After each 24‐h period, specimens were rinsed with distilled water for 5 min, dried, and, after 7 days, final color measurements were performed.

Color differences were calculated using the CIEDE2000 formula (Δ*E*00), adopting parametric factors KL = KC = KH = 1 (Sharma et al. 2004). A Δ*E*00 value > 1.30 was considered clinically perceptible, and values ≤ 2.25 were considered clinically acceptable. Additionally, Δ*E* values were converted into National Bureau of Standards (NBS) units (NBS = Δ*E* × 0.92) to classify the magnitude of color change (Ren et al. 2015). According to established thresholds, Δ*E* values between 0 and 2 is considered imperceptible, 2–3 slightly perceptible (Seghi et al. 1990), and values ≥ 3.3 clinically unacceptable (Gross and Moser 1977).

$$\Delta E_{00}=\sqrt{\left[{\left(\frac{\Delta L}{K_{L}S_{L}}\right)}^{2}+{\left(\frac{\Delta C}{K_{C}S_{C}}\right)}^{2}+{\left(\frac{\Delta H}{K_{H}S_{H}}\right)}^{2}+R_{T}\left(\frac{\Delta C}{K_{C}S_{C}}\right)\left(\frac{\Delta H}{K_{H}S_{H}}\right)\right]}$$

### 2.6 Bacterial Adhesion

The standard strain *Streptococcus mutans* (ATCC) was cultured in Brain Heart Infusion (BHI) broth. Inoculum standardization and biofilm quantification were performed according to Stepanovic et al. (2007), with modifications.

After 48 h of incubation for biofilm formation, specimens were washed twice with sterile saline to remove non‐adherent cells. Biofilm detachment was performed by ultrasonic treatment, followed by centrifugation at 3000 rpm for 15 min to obtain the biofilm pellet. The pellet was dried, and dry weight was determined.

Biofilm biomass was also assessed by crystal violet staining. After staining for 15 min and removal of excess dye, the bound dye was resolubilized with 95% ethanol. Absorbance was measured using a 96‐well microplate reader.

### 2.7 Statistical Analysis

Sample size power was calculated using OpenEpi software, adopting a 95% confidence interval. Data normality was assessed using the Shapiro–Wilk test in SPSS (IBM SPSS Statistics 22 for Windows, SPSS Inc., Chicago, IL, USA). Surface roughness (*p* < 0.001) and color stability in distilled water (*p* = 0.007) did not demonstrate normal distribution and were therefore analyzed using the nonparametric Kruskal–Wallis test for intergroup comparisons. Intragroup comparisons were performed using the Wilcoxon signed‐rank test. Wettability and bacterial adhesion data showed normal distribution and were analyzed by one‐way analysis of variance (ANOVA) followed by Tukey's post hoc test. The level of significance was set at *α* = 0.05.

## 3 Results

### 3.1 Surface Roughness

Table 2 presents the average surface roughness values (*R*_a_, µm) obtained from the 60 samples. The bis‐acryl composite resin (BCR) exhibited the lowest roughness values, being significantly lower than the other materials (*p* < 0.001), while the prefabricated acrylic tooth (PT) presented the highest values. Figure 3 shows the three‐dimensional optical profilometry images of the evaluated groups, evidencing a progressive increase in surface roughness from the BCR group to the PT group.

**Table 2:** Surface roughness values (*R*_a_, µm) for the experimental groups.

Groups	Surface roughness (*R*_a_) (median ± Q25–Q75)
BCR	0.08 ± (0.03–0.15)^A^
Milled	0.19 ± (0.14–0.80)^B^
APR	0.21 ± (0.13–0.27)^B^
PT	0.33 ± (0.20–1.33)^B^

*Note:* Different superscripted capital letters in the same column represent statistically significant differences (*p* < 0.001—Kruskal–Wallis test).

Abbreviations: APR, Auto‐polymerized acrylic resin; BCR, bis‐acryl composite resin; Milled, CAD‐CAM PMMA resin; PT, prefabricated acrylic tooth.

**Figure 3:**
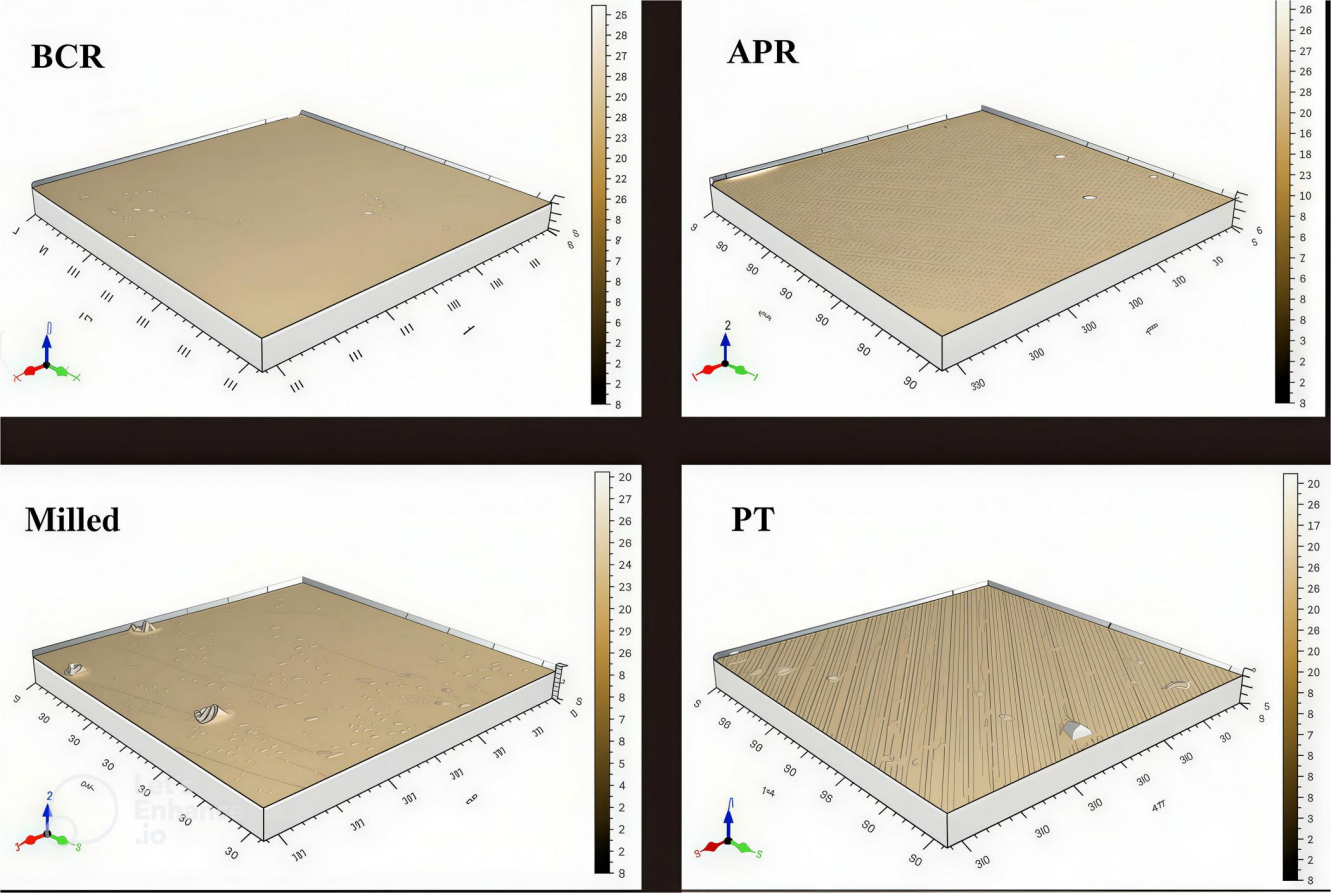
3D image obtained by profilometry of the surface of the samples for each group. APR, Auto‐polymerized acrylic resin; BCR, bis‐acryl composite resin; Milled, CAD‐CAM PMMA resin; PT, prefabricated acrylic tooth.

### 3.2 Wettability

One‐way ANOVA revealed a significant effect of material type on contact angle values (Table 3). The prefabricated acrylic tooth (PT) exhibited a significantly higher contact angle than the bis‐acryl composite resin (BCR) (*p* < 0.05), indicating lower wettability. The CAD‐CAM PMMA resin (Milled) and Auto‐polymerized acrylic resin (APR) showed intermediate values, without statistically significant differences from either PT or BCR. Figure 4 illustrates the representative contact angle measurements between distilled water and the surface of each experimental group.

**Table 3:** Mean ± standard deviation (SD) of contact angle (*Ѳ*) values for tested materials.

Groups	Contact angle (*Ѳ*) (mean ± standard)
BCR	65.01 ± (3.86)^A^
Milled	72.71 ± (5.60)^AB^
APR	71.64 ± (4.61)^AB^
PT	76.58 ± (8.37)^B^

*Note:* Values whose uppercase superscript letters differ from each other in the same column represent statistically significant differences between them (*p* < 0.01—one‐way ANOVA, Tukey's post‐test (5%).

Abbreviations: APR, Auto‐polymerized acrylic resin; BCR, bis‐acryl composite resin; Milled, CAD‐CAM PMMA resin; PT, prefabricated acrylic tooth.

**Figure 4:**
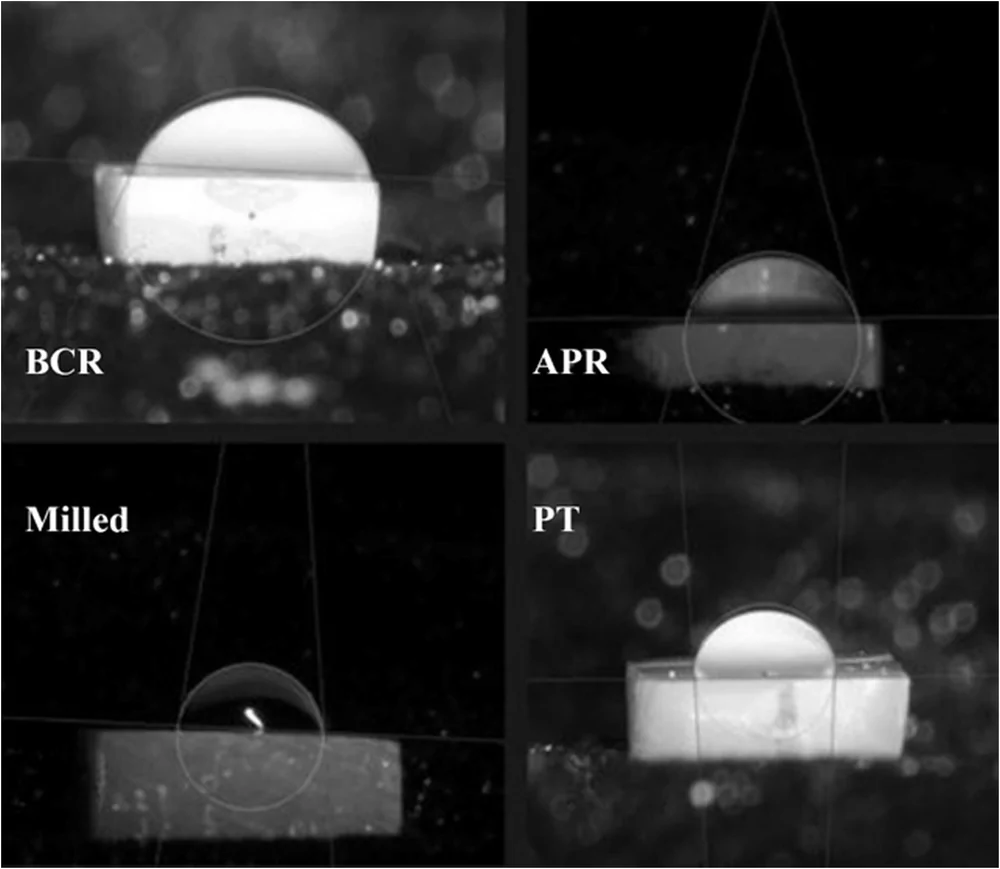
Aspect obtained using Surftens software, forming the contact angle of the droplet with the surface of the samples from each group. APR, Auto‐polymerized acrylic resin; BCR, bis‐acryl composite resin; Milled, CAD‐CAM PMMA resin; PT, prefabricated acrylic tooth.

### 3.3 Color Stability Analysis

Color stability analysis demonstrated significant differences among materials after 7 days of immersion (Table 4). The exhibited the highest Δ*E* values in both distilled water and coffee (Δ*E* ≥ 3.3), corresponding to clinically unacceptable discoloration. In distilled water, PT showed markedly higher color change according to the NBS criteria, and its Δ*E* was significantly greater than that of the Auto‐polymerized acrylic resin (APR) (*p* = 0.015). Following coffee immersion, both the Bis‐acryl composite resin (BCR) and PT demonstrated excessively marked discoloration. APR was the only material that remained within clinically acceptable limits. The Δ*E* of PT was significantly higher than that of APR (*p* = 0.049) and CAD‐CAM PMMA resin (*p* = 0.032). Intergroup analysis revealed an increase in Δ*E* values for all materials after 7 days, with greater discoloration observed in coffee compared with distilled water. However, a statistically significant difference between storage media was detected only for BCR (*p* = 0.028).

**Table 4:** Color stability after 7 days of immersion in distilled water and coffee.

Groups	Distilled water (median ± Q25–Q75)	Coffee (median ± Q25–Q75)
BCR	2.27 ± (1.91–3.33)^ABa^	7.31 ± (4.95–7.74)^CDb^
Milled	2.80 ± (0.66–10.37)^ABc^	4.61 ± (2.35–6.37)^Cc^
APR	1.67 ± (1.48–3.63)^Bd^	2.18 ± (2.07–9.78)^Cd^
PT	7.96 ± (7.51–8.16)^Ae^	8.92 ± (7.84–10.78)^De^

*Note:* Different superscripted capital letters in the same column represent statistically significant differences (*p* < 0.05). Different superscripted lowercase letters in the row indicate statistically significant differences. Tests used: Kruskal–Wallis test.

Abbreviations: APR, Auto‐polymerized acrylic resin; BCR, bis‐acryl composite resin; Milled, CAD‐CAM PMMA resin; PT, prefabricated acrylic tooth.

### 3.4 Bacterial Adhesion

Biofilm formation differed significantly among the materials (one‐way ANOVA, *p* < 0.0001). The bis‐acryl composite resin (BCR) exhibited the highest mean biofilm formation (75.28 ± 2.97), significantly greater than all other groups. In contrast, the CAD‐CAM PMMA resin (Milled) showed the lowest bacterial adhesion (57.65 ± 5.25). The Auto‐polymerized acrylic resin (APR) (64.43 ± 2.88) and the prefabricated acrylic tooth (PT) (68.38 ± 1.40) demonstrated intermediate values, without significant differences between them (Table 5). Representative colorimetric analysis of biofilm formation is presented in Figure 5, illustrating higher biofilm accumulation in the BCR group compared with the other materials.

**Table 5:** Mean ± standard deviation (SD) of microorganism adhesion for the experimental groups.

Groups	Biofilm formation (mean ± standard)
BCR	75.28 ± (2.97)^C^
Milled	57.65 ± (5.25)^A^
APR	64.43 ± (2.88)^B^
PT	68.38 ± (1.40)^B^

*Note:* Different capital letters indicate statistical differences between the groups (*p* < 0.0001) ‐ ANOVA ‐ 1 factor, Tukey's post‐test (5%).

Abbreviations: APR, Auto‐polymerized acrylic resin; BCR, bis‐acryl composite resin; Milled, CAD‐CAM PMMA resin; PT, prefabricated acrylic tooth.

**Figure 5:**
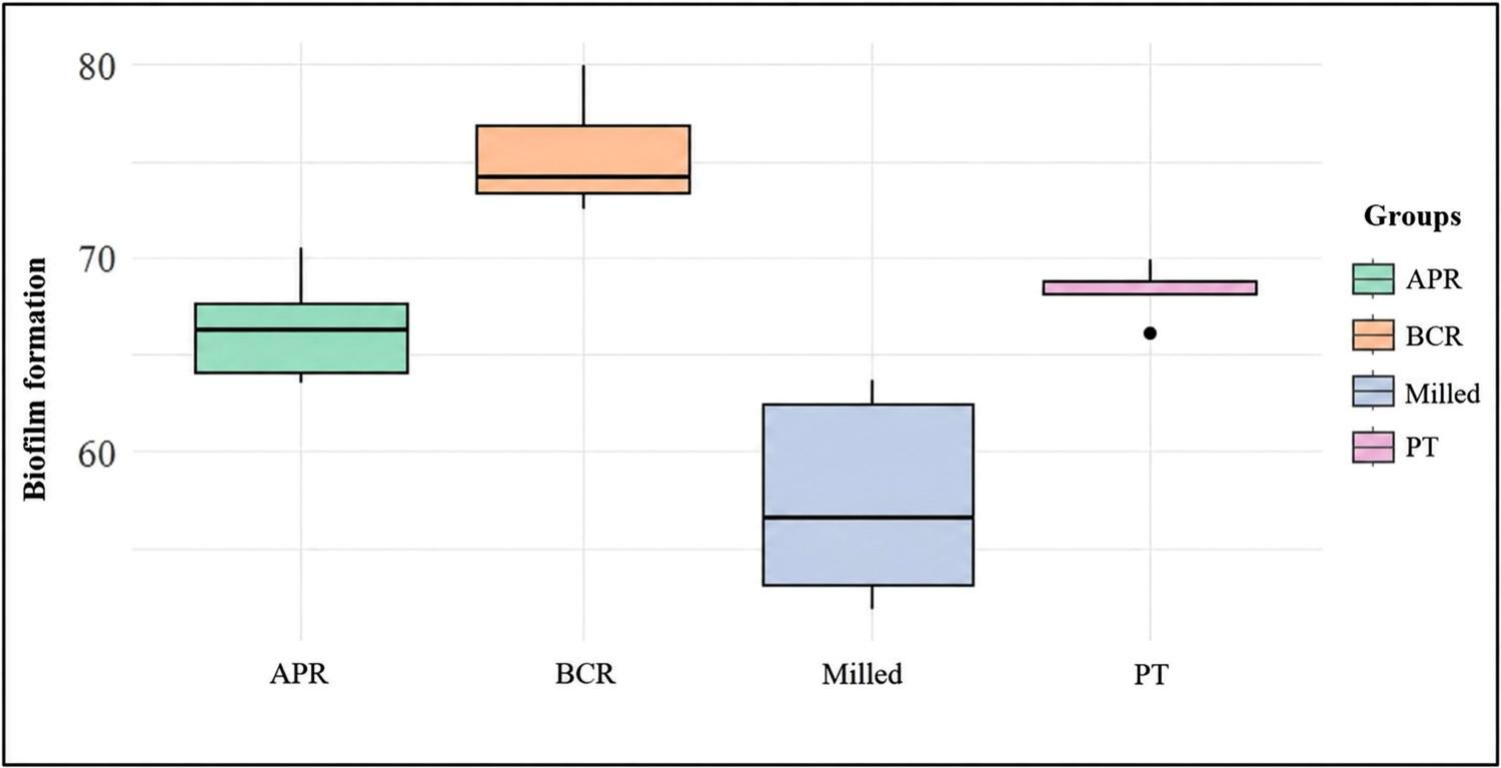
Colorimetric analysis of biofilm formation in experimental groups. APR, Auto‐polymerized acrylic resin; BCR, bis‐acryl composite resin; Milled, CAD‐CAM PMMA resin; PT, prefabricated acrylic tooth.

## 4 Discussion

The present study provides an assessment of the surface behavior of different materials used for implant‐supported provisional restorations, addressing an important clinical question given the increasingly prolonged use of provisional restorations during peri‐implant soft‐tissue conditioning in esthetic rehabilitation. The findings demonstrated that material composition can play a role in these properties but also revealed that their behavior cannot be predicted by surface characteristics alone. Notably, the bis‐acryl composite resin exhibited the lowest surface roughness yet showed the highest bacterial adhesion, whereas the prefabricated acrylic tooth demonstrated clinically unacceptable color change. These findings challenge the assumption that smoother surfaces necessarily result in lower bacterial accumulation or superior overall performance and indicate that intrinsic material characteristics may influence biological and esthetic outcomes. Thus, the study hypothesis that the different materials would exhibit significant differences in surface properties was partially accepted.

Results demonstrated a significantly lower surface roughness for the bis‐acryl composite resin compared to the other materials. Previous studies reported roughness values ranging from 0.07 to 1.27 μm for bis‐acrylic materials (Soares et al. 2019; Schwantz et al. 2017; Tupinamba et al. 2018), corroborating the smoother surface observed in this study. This behavior can be attributed to the presence of filler nanoparticles and the automatic dosing system, which provides more precise proportions, reduces air entrapment, and minimizes polymerization shrinkage and exothermic reaction (Gantz et al. 2021; Soares et al. 2019). Wettability is another relevant factor in the biological behavior of restorative materials, as higher contact angles are generally associated with reduced solid‐liquid interaction and potentially lower microbial attachment (Wu et al. 2018; Kreve and dos Reis 2021). In the present study, the higher contact angle observed for PT indicates lower liquid affinity; however, when considered alongside the surface roughness findings, it becomes clear that wettability alone does not influence microbial behavior (Gantz et al. 2021; Haralur et al. 2024).

Although previous studies have reported greater discoloration for conventional bis‐acrylic and PMMA resins (Elagra et al. 2017; Almohareb et al. 2018), this study found that prefabricated acrylic tooth (PT) exhibited the highest Δ*E* values, while Auto‐polymerized acrylic resin maintained acceptable stability. The unsatisfactory performance of PT may be attributed to the removal of the outermost vestibular layer during sample preparation. This process likely exposed the inner layers, which often contain potentially hydrophilic components, which may have facilitated the staining (Motayagheni et al. 2020). Although bis‐acryl composite resin showed pronounced staining after coffee immersion and was the only material significantly affected by the storage medium (*p* = 0.028) this behavior may be attributed to its higher diffusion coefficient and water sorption (Elagra et al. 2017). The presence of hydrophilic Bis‐GMA in its matrix, capable of hydrogen bonding with water, as well as its heterogeneous polymer network, may further facilitate pigment penetration and retention (Sánchez‐Tito et al. 2023; Shaikh et al. 2025). The chemical stability of APR allowed it to maintain the lowest color change, suggesting that its homogeneous PMMA matrix is less susceptible to pigment infiltration (Soares et al. 2019).

Based on the results obtained, a relationship can be inferred between surface wettability and bacterial adhesion. The Bis‐acryl composite resin presented the lowest contact angle values, indicating greater surface hydrophilicity, and simultaneously presented the highest levels of biofilm formation. These results are consistent with previous evidence indicating that the microbiota of the oral environment has high free energy and preferentially adheres to hydrophilic substrates (Kreve and dos Reis 2021; Mazurek‐popczyk et al. 2022). The superior biological performance of CAD‐CAM PMMA resin may be attributed to its high degree of conversion and industrial polymerization, which minimizes micro‐porosities available for bacterial colonization (Simoneti et al. 2022).

In summary, the present findings demonstrate that the type of provisional restorative material influences its surface behavior, with surface characteristics contributing to differences in color stability and biofilm formation. However, limitations such as the absence of aging, the use of a single finishing protocol, and the need for vestibular wear of prefabricated teeth should be considered. Although the experimental conditions did not reproduce all aspects of the dynamic oral environment, the standardized protocol allowed a controlled comparison of the materials and provided relevant evidence regarding their intrinsic properties. Further investigations incorporating artificial aging, repeated clinical simulation, and complementary cellular and biofilm models, including in situ approaches, may expand these findings and provide additional insight into the long‐term biological and esthetic performance of implant‐supported provisional restorative materials.

## 5 Conclusion

Within the limitations of this in vitro study, the composition of implant‐supported provisional restorative materials significantly influenced their surface, optical, and biological behavior. CAD/CAM PMMA resin demonstrated the most favorable overall profile, particularly regarding bacterial adhesion. In contrast, prefabricated acrylic teeth exhibited the least favorable behavior, characterized primarily by clinically unacceptable color instability and less favorable surface characteristics. Bis‐acryl composite resin presented the lowest surface roughness but was associated with greater color alteration and bacterial adhesion, highlighting that a smoother surface does not necessarily translate into superior biological or esthetic performance. Autopolymerized acrylic resin demonstrated acceptable color stability under the conditions evaluated. These findings support the importance of considering the combined surface, optical, and biological characteristics of provisional restorative materials when selecting materials for implant‐supported provisional restorations, particularly when prolonged provisionalization and esthetic stability are required.

## Author Contributions

**Júlia Bárbara Medeiros Galvão:** investigation, data acquisition, and writing – original draft. **Richelle Nunes Costa:** investigation, data acquisition, and writing – original draft. **Aliane da Silva Bezerra:** data analysis and writing – review and editing. **Liliane Cristina Nogueira Marinho:** investigation, supervision and writing – original draft. **Ana Luísa de Barros Pascoal:** data analysis. **Pedro Henrique Sette de Souza:** methodology, investigation and data curation. **André Ulisses Dantas Batista:** methodology, investigation and data curation. **Patrícia dos Santos Calderon:** conceptualization, supervision, project administration and writing – review and editing.

## Conflicts of Interest

The authors declare no conflicts of interest.

## Data Availability

The data that support the findings of this study are available from the corresponding author upon request.
